# Causal relationship between birth weight, uric acid levels, and risk of gout: New insights from a bidirectional two-sample Mendelian randomization study

**DOI:** 10.1097/MD.0000000000049237

**Published:** 2026-06-05

**Authors:** Meile Yin, Yulai Yin, Huiying Feng

**Affiliations:** a Neonatal Intensive Care Unit, Chengde Central Hospital, Chengde, China.

**Keywords:** birth weight, genetics, gout, Mendelian randomization, uric acid

## Abstract

Bidirectional two-sample Mendelian randomization (MR) was used to evaluate the potential causal effects of birth weight and uric acid levels (the exposures) and the development of gout (the outcome). Genetic instruments derived from aggregated genome-wide association study data of European ancestry were used to investigate the associations between birth weight (261,932 samples, 9,851,867 single-nucleotide polymorphisms [SNPs]), uric acid levels (110,347 samples, 4,865,122 SNPs), and gout (337,159 samples, including 4807 cases and 332,352 controls, 10,894,596 SNPs). The inverse variance weighting method was used for primary causal estimates, and a series of sensitivity analyses were performed to assess the robustness of the results. The study found that birth weight was negatively associated with uric acid levels (odds ratio [OR] = 0.849; 95% confidence interval [CI]: 0.786–0.917; *P* < .001) and correspondingly had a negative impact on gout risk (OR = 0.997; 95% CI: 0.994–0.9997; *P* = .032). In reverse MR analyses, genetic liability for gout was strongly associated with lower birth weight (OR = 0.380; 95% CI: 0.222–0.650; *P* < .001). This indicates that individuals with a higher genetic predisposition to gout have 62% lower odds of having a higher birth weight. However, the reverse MR analysis of birth weight and uric acid levels was influenced by horizontal pleiotropy and could not provide additional scientific insights. Genetically predicted higher birth weight demonstrated a protective causal effect on serum urate levels. Specifically, each 1-standard deviation increase in birth weight was associated with a 15.1% reduction in the odds of hyperuricemia (OR = 0.849; 95% CI: 0.786–0.917; *P* < .001). This causal estimate was robust across sensitivity analyses, including the weighted median and MR-Egger methods, with no evidence of directional pleiotropy (Egger intercept *P* > .05). Leave-one-out analysis confirmed that the results were not driven by any single influential genetic variant.

## 1. Introduction

Gout^[1,2]^ is an inflammatory arthritis triggered by the deposition of monosodium urate crystals in the joints and surrounding tissues. It is characterized by acute episodes of joint pain, swelling, and redness, primarily presenting as recurrent acute arthritis that most commonly affects the lower limbs, especially the first metatarsophalangeal joint. The onset of gout is associated with various factors, including genetics, diet, sex, and age. In developed countries, it is the most prevalent form of inflammatory arthritis and is typically linked to elevated serum urate levels (hyperuricemia). When serum urate concentrations exceed the solubility threshold (typically 6.8 mg/dL),^[3–5]^ uric acid crystallizes in the joints and tissues, forming monosodium urate crystals. These crystals induce a localized immune response, activating immune cells such as neutrophils and macrophages, which release inflammatory mediators and generate an inflammatory response. This process leads to the characteristic symptoms of acute arthritis, such as severe pain, swelling, redness, and localized temperature elevation. Globally, the prevalence of gout is increasing, particularly in developed nations. Studies indicate that gout prevalence in Western countries ranges from 1% to 4%, with men significantly more affected than women. The condition primarily affects older men and is more common in individuals over 40. Recent increases in gout prevalence among younger populations have also been noted due to the rise in obesity and metabolic syndrome. Therefore, monitoring and controlling serum urate levels is essential for preventing the onset of gout.

Birth weight^[6,7]^ refers to the weight of a newborn at birth and is a critical indicator of fetal growth and development. It is closely linked to neonatal health and the long-term health of children and adults. Typically, full-term newborns weigh between 2500 and 4000 grams. Newborns weighing less than 2500 grams are classified as low birth weight (LBW), while those weighing over 4000 grams are classified as macrosomic. LBW can be further divided into very LBW, defined as less than 1500 grams, and extremely LBW, which is less than 1000 grams. Globally, the proportion of LBW infants is significant, especially in developing countries, where factors such as malnutrition, inadequate prenatal care, and maternal infections contribute to high LBW incidence. This condition increases the risk of neonatal mortality and childhood diseases. Birth weight is influenced by various factors, including maternal nutrition, smoking, and alcohol consumption, pregnancy infections, placental function, and pregnancy-related conditions such as hypertension and diabetes. LBW is linked to increased risks of early infant mortality, neurodevelopmental abnormalities, and chronic diseases like diabetes and hypertension, while macrosomia is associated with obstetric complications and a higher risk of obesity and metabolic diseases. Overall, birth weight serves as an early health indicator and holds significant importance for research on child development, disease prevention, and public health policy.

Mendelian randomization (MR),^[8–11]^ primarily based on single-nucleotide polymorphisms (SNPs), infers causal relationships between exposure and disease outcomes through genetic variation. In MR studies, genetic variations associated with phenotypes serve as instrumental variables (IVs) for exposure, enabling causal inference between exposure and outcome. Genetic variation follows the principle of random segregation of alleles from parents to offspring and is determined at conception; therefore, it is less prone to confounding factors present in traditional observational studies. Consequently, this study employs a bidirectional two-sample MR analysis to explore the causal relationship between birth weight, serum urate levels, and gout.

## 2. Materials and methods

**Figure 1.**
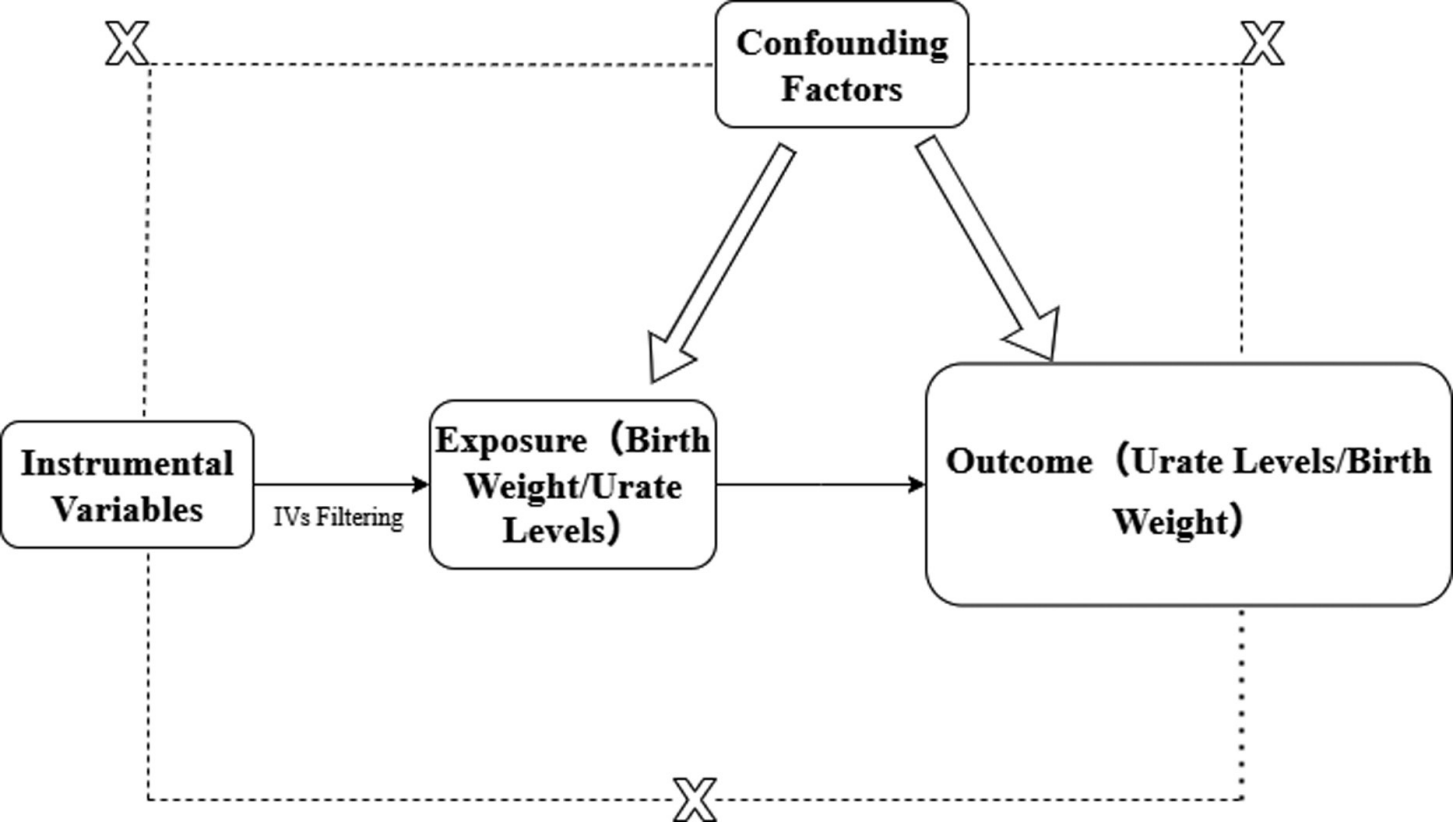
Principle of instrumental variable selection. IV = instrumental variable.

For MR studies to yield effective and reliable conclusions, IV selection must satisfy 3 core assumptions: genetic variation must be strongly correlated with exposure; it must affect the outcome exclusively through the exposure rather than directly or through alternative pathways; and it must not be linked to any confounding factors influencing the exposure–outcome relationship (Fig. 1). To minimize the impact of linkage disequilibrium,^[12,13]^ we chose SNPs passing the genome-wide significance threshold (*P* < 5 × 10^−8^, *r*^2^ ≤ 0.001, meeting Hardy–Weinberg equilibrium, and genetic distance <10,000 kb) as IVs. The *F*-statistic was calculated for each IV to ensure only those with *F* > 10 were included, avoiding bias from weak instruments. To analyze the causal relationships between exposures (birth weight and serum urate) and outcomes (gout), 156 and 139 SNPs were chosen, respectively. For reverse MR analyses, 27 SNPs linked to serum urate and 21 SNPs linked to gout were selected.

For birth weight, summary statistics from a genome-wide association study (GWAS) comprising 261,932 samples and 9,851,867 SNPs from individuals of European descent were used. For serum urate levels, GWAS summary statistics included 110,347 samples and 4,865,122 SNPs of European descent. For gout, summary statistics from GWAS included 337,159 samples (4807 cases and 332,352 controls) and 10,894,596 SNPs from individuals of European ancestry.

## 3. Data availability

The GWAS summary statistics for birth weight, serum urate, and gout were sourced from the OpenGWAS database (https://gwas.mrcieu.ac.uk/). Relevant SNP data are detailed in Tables S1 to S8, Supplemental Digital Content 1–8.

## 4. Statistical analysis

The MR analyses were based on summary-level statistics (aggregated data from GWAS) and not individual-level data. The MR analyses can be considered a type of meta-analysis that combines results from GWAS data.

The inverse variance weighted (IVW) method was the primary analysis technique for MR, supplemented by Cochran *Q* and Rucker *Q* heterogeneity tests via IVW and MR-Egger. Pleiotropy was evaluated with the Egger intercept test, and sensitivity analyses were conducted via the leave-one-out method.

To strengthen the evidence base, an independent MR study using the PhenoScanner database excluded pleiotropic SNPs confounded by exposure–outcome associations.

### 4.1. Handling of missing data and potential selection bias

The MR analyses were performed using summary-level data from large-scale GWAS; therefore, individual-level missing data were handled at the primary study level, typically through complete-case analysis within the original cohorts. We minimized potential bias from missing IVs by ensuring that all selected SNPs were available in both the exposure and outcome datasets.

Effect estimates are expressed as adjusted odds ratios (ORs) with 95% confidence intervals (CIs). For multiple testing, associations with a *P*-value below the Bonferroni-corrected α = 0.0125 were considered statistically significant, while those with *P* ≥ .0125 and <.05 were considered suggestively significant. All analyses were conducted using the open-source statistical software R (version 4.3.2; The R Foundation for Statistical Computing), the MR analysis website (http://app.mrbase.org/), OpenGWAS (https://gwas.mrcieu.ac.uk/), and the PhenoScanner website (http://www.phenoscanner.medschl.cam.ac.uk/). The report follows the STROBE-MR guidelines^[14,15]^ (Strengthening the Reporting of Observational Studies in Epidemiology–MR).

### 4.2. Sensitivity analysis

Cochran *Q* and Rucker *Q* heterogeneity tests were performed using IVW and MR-Egger methods (*P* > .05 indicating heterogeneity). If heterogeneity was present, the Mendelian randomization pleiotropy residual sum and outlier method was employed with 1000 distributions to remove outliers. The Egger intercept pleiotropy tests showed no evidence of pleiotropy (*P* > .05). All MR analysis results presented herein show no evidence of horizontal pleiotropy, and Mendelian randomization pleiotropy residual sum and outlier results confirm statistical significance.

## 5. Ethical statement

This study utilized publicly available GWAS databases. All original studies obtained ethical approval.

## 6. Results

**Figure 2.**
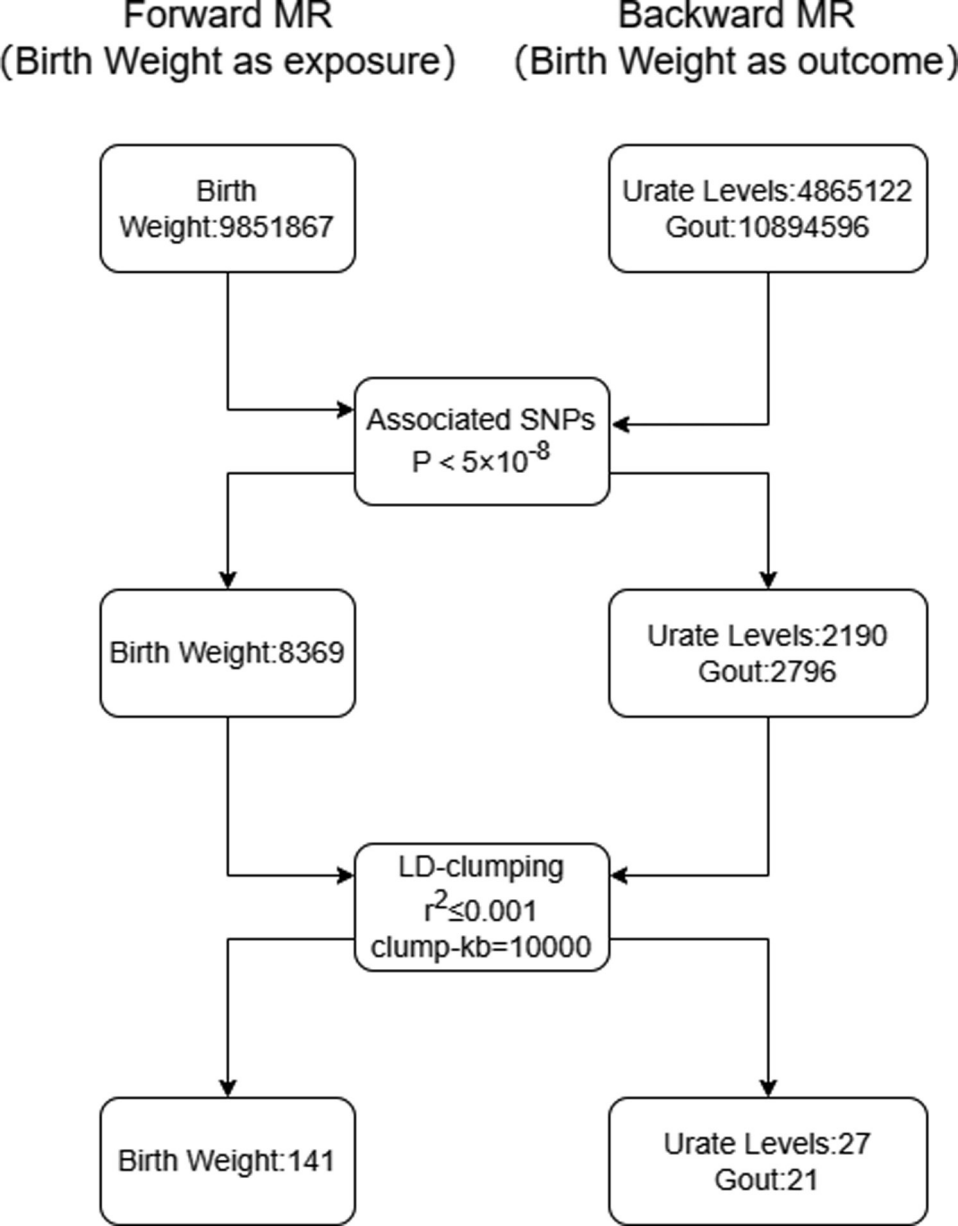
Selection process for SNPs in Mendelian randomization analysis. The figure is divided into 2 columns, each illustrating the step-by-step selection of SNPs utilized in MR. The left column delineates the process for forward MR with birth weight as the exposure. The initial step involves the exclusion of SNPs with association *P*-values ≥ 5 × 10^−8^. Subsequent refinement eliminates SNPs exhibiting linkage disequilibrium with *r*^2^ > 0.001 or located within 10,000 kb of each other. Each step lists the remaining number of SNPs after filtration. Conversely, the right column describes the selection procedure for reverse MR, where birth weight serves as the outcome variable. The sequential SNP filtering criteria and the corresponding residual SNP counts are similarly detailed, ensuring clarity in the depiction of the analytic rigor applied. LD = linkage disequilibrium, MR = Mendelian randomization, SNPs = single-nucleotide polymorphisms.

**Figure 3.**
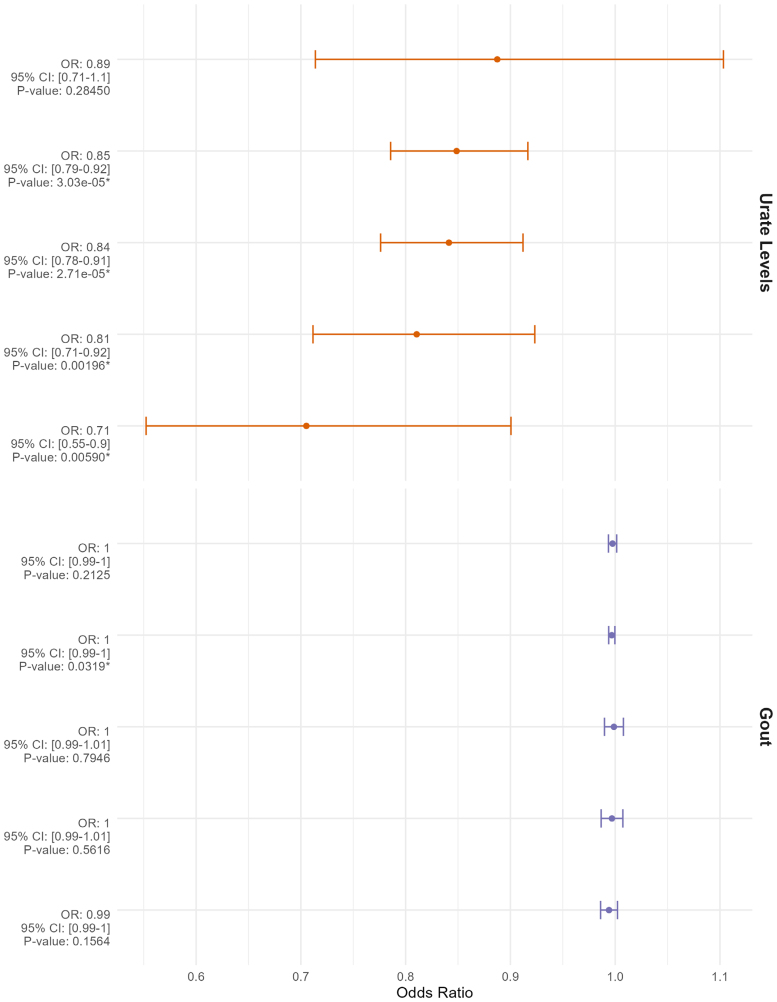
Mendelian randomization analysis of birth weight impact on urate levels and gout. This figure sequentially presents the results of MR analysis where birth weight is considered as the exposure, with serum urate levels and gout as outcomes. The findings are quantified using adjusted ORs and 95% CIs. From top to bottom, the MR methodologies applied include “MR Egger,” “Weighted Median,” “Inverse Variance Weighted,” “Simple Mode,” and “Weighted Mode.” Each method provides a distinct analytical approach to ascertain the causal inference between the genetic predisposition to higher birth weight and subsequent health outcomes. A symbol “*” indicates statistically significant results where *P* < .05, emphasizing findings where genetic instruments strongly suggest a causal relationship. This structured presentation allows for a comprehensive assessment of the robustness and consistency of the causal links observed. CIs = confidence intervals, MR = Mendelian randomization, ORs = odds ratios.

**Figure 4.**
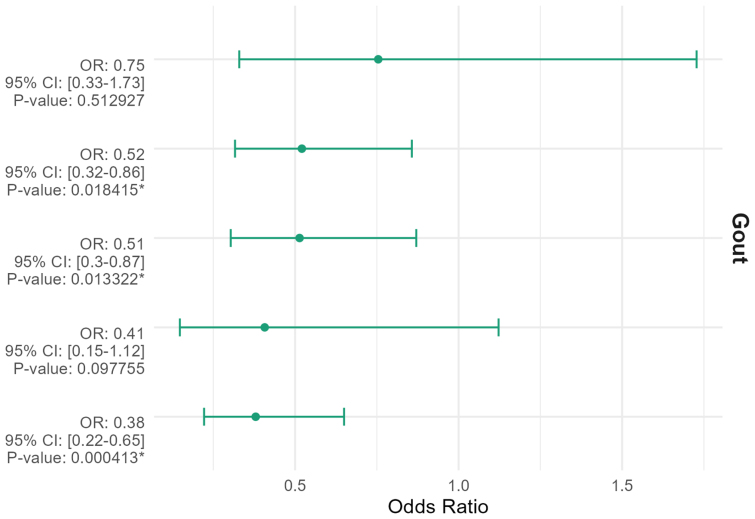
Mendelian randomization analysis of gout impact on birth weight levels. The figure displays the results of MR analysis with gout as the exposure and birth weight levels as the outcome. The outcomes are represented using adjusted ORs and 95% CIs. From top to bottom, the MR methodologies applied are “MR Egger,” “Weighted Median,” “Inverse Variance Weighted,” “Simple Mode,” and “Weighted Mode.” Each method offers a different statistical approach to assess the potential causal influence of genetic predisposition to gout on birth weight. The symbol “*” indicates statistical significance where *P* < .05, underscoring results that strongly suggest a causal linkage. This orderly presentation aids in evaluating the strength and consistency of the hypothesized causal relationships. CIs = confidence intervals, MR = Mendelian randomization, ORs = odds ratios.

The SNP selection process is detailed in Figure 2. Estimates obtained via IVW are reported here. Genetically predicted birth weight was negatively correlated with serum urate levels (OR = 0.849; 95% CI: 0.786–0.917; *P* = 3.03E−5; Fig. 3). Similarly, genetically predicted birth weight was negatively associated with gout (OR = 0.997; 95% CI: 0.994–0.9997; *P* = .032; Fig. 3). In reverse MR analyses, genetic liability for gout was strongly associated with lower birth weight (OR = 0.380; 95% CI: 0.222–0.650; *P* < .001). This indicates that individuals with a higher genetic predisposition to gout have 62% lower odds of having a higher birth weight (Fig. 4). However, the reverse MR analysis of birth weight and serum urate showed pleiotropy (*P* < .05), limiting the scientific contribution.

All MR analyses employed the Egger intercept test to assess pleiotropy, with no evidence observed (*P* > .05), except in the reverse MR analysis between birth weight and serum urate.

## 7. Discussion

Our study reveals a negative correlation between genetically predicted birth weight levels and both serum urate levels and the risk of developing gout, suggesting that serum urate levels may, in part, mediate the influence on the incidence of gout. Conversely, reverse MR analysis indicates a robust negative correlation between genetically predicted gout and birth weight levels, although the reverse causal relationship between birth weight and serum urate levels is confounded by pleiotropy, leading to less consistent conclusions. The consistency between the bidirectional two-sample MR analyses for birth weight and gout further robustly confirms the negative causal relationship between birth weight levels and the risk of gout. Additionally, the impact of birth weight levels on serum urate levels, compared with its impact on the incidence of gout, shows a more significant OR, further suggesting that birth weight may indirectly influence the onset of gout by affecting urate levels.

Cohort studies have shown that fetal birth weight is closely related to the maternal diet during pregnancy.^[16–18]^ Extensive research has also demonstrated the correlation between birth weight and the incidence of various diseases. For instance, a longitudinal cohort study by Eriksson et al^[19]^ followed 478 men born in 1913 from age 50 to 80, finding that LBW increased the incidence of hypertension, diabetes, and hypercholesterolemia. In the general population, the highest risk percentage for diabetes is seen in individuals with a birth weight ≤3000 grams. Geng et al’s prospective cohort study^[20]^ further confirmed that intrauterine growth restriction could alter epigenetic characteristics, particularly the histone code at the liver insulin-like growth factor 1 (*IGF-1*) gene, which disrupts the epigenetics of development around the distal growth hormone response elements on the liver *IGF-1* gene, disrupting the formation of the nucleosome-depleted region on the right side of the *IGF-1* gene, leading to a sustained reduction in serum *IGF-1* and thus impacting glucose tolerance and promoting diabetes. Moreover, studies indicate that birth weight is positively related to bone density during adolescence,^[21]^ suggesting that fetal development may influence metabolism, which in turn affects the development of bones and muscles, potentially contributing to diseases such as osteoporosis and sarcopenia. Beyond nonneoplastic diseases, a cohort study by Spracklen et al^[22]^ explored the relationship between birth weight and the incidence of neoplastic diseases. Their findings indicated significant associations between birth weight categories and the risk of postmenopausal cancers, though the direction of the associations varied by cancer type, with birth weight positively correlated with the risks of lung cancer (*P* = .01) and colon cancer (*P* = .04), and inversely correlated with the risk of leukemia (*P* = .04).

## 8. Strengths and limitations of the study

This study’s strength lies in its novel use of two-sample MR to explore the bidirectional causal relationship between birth weight, serum urate levels, and gout incidence. Unlike observational studies, our approach is akin to a randomized controlled trial and is less susceptible to confounding factors, providing a higher level of evidence. Additionally, the use of the Egger intercept for assessing pleiotropy confirms the reliability of our results.

The limitations of this study include its reliance on data from European populations, which may limit the generalizability of our findings across different ethnicities. Additionally, the lack of data granularity restricts further stratified statistical analysis of birth weight. The absence of validation from other databases might introduce bias into our conclusions.

## 9. Conclusion

Genetically predicted higher birth weight demonstrated a protective causal effect on serum urate levels. Specifically, each 1-standard deviation increase in birth weight was associated with a 15.1% reduction in the odds of hyperuricemia (OR = 0.849; 95% CI: 0.786–0.917; *P* < .001). This causal estimate was robust across sensitivity analyses, including the weighted median and MR-Egger methods, with no evidence of directional pleiotropy (Egger intercept *P* > .05). Leave-one-out analysis confirmed that the results were not driven by any single influential genetic variant. The detailed and specific MR analysis results are visualized in Figures S1 to S14, Supplemental Digital Content 2–14.

## Acknowledgments

We thank Dr Yulai Yin for his outstanding contribution to this article.

## Author contributions

**Conceptualization:** Huiying Feng.

**Methodology:** Yulai Yin.

**Data curation:** Huiying Feng.

**Software:** Meile Yin.

**Validation:** Meile Yin, Yulai Yin.

**Visualization:** Meile Yin, Yulai Yin.

**Writing – original draft:** Meile Yin, Yulai Yin.

**Writing – review & editing:** Meile Yin, Yulai Yin, Huiying Feng.












































