# Supplementary figures and images for: Causal relationship between birth weight, uric acid levels, and risk of gout: New insights from a bidirectional two-sample Mendelian randomization study
Source: Medicine (Baltimore). 2026 Jun 5;105(23):e49237. doi: 10.1097/MD.0000000000049237 (PMC13246114; doi:10.1097/MD.0000000000049237)

# MR Test

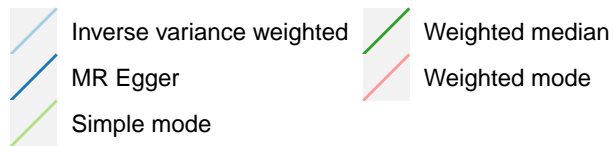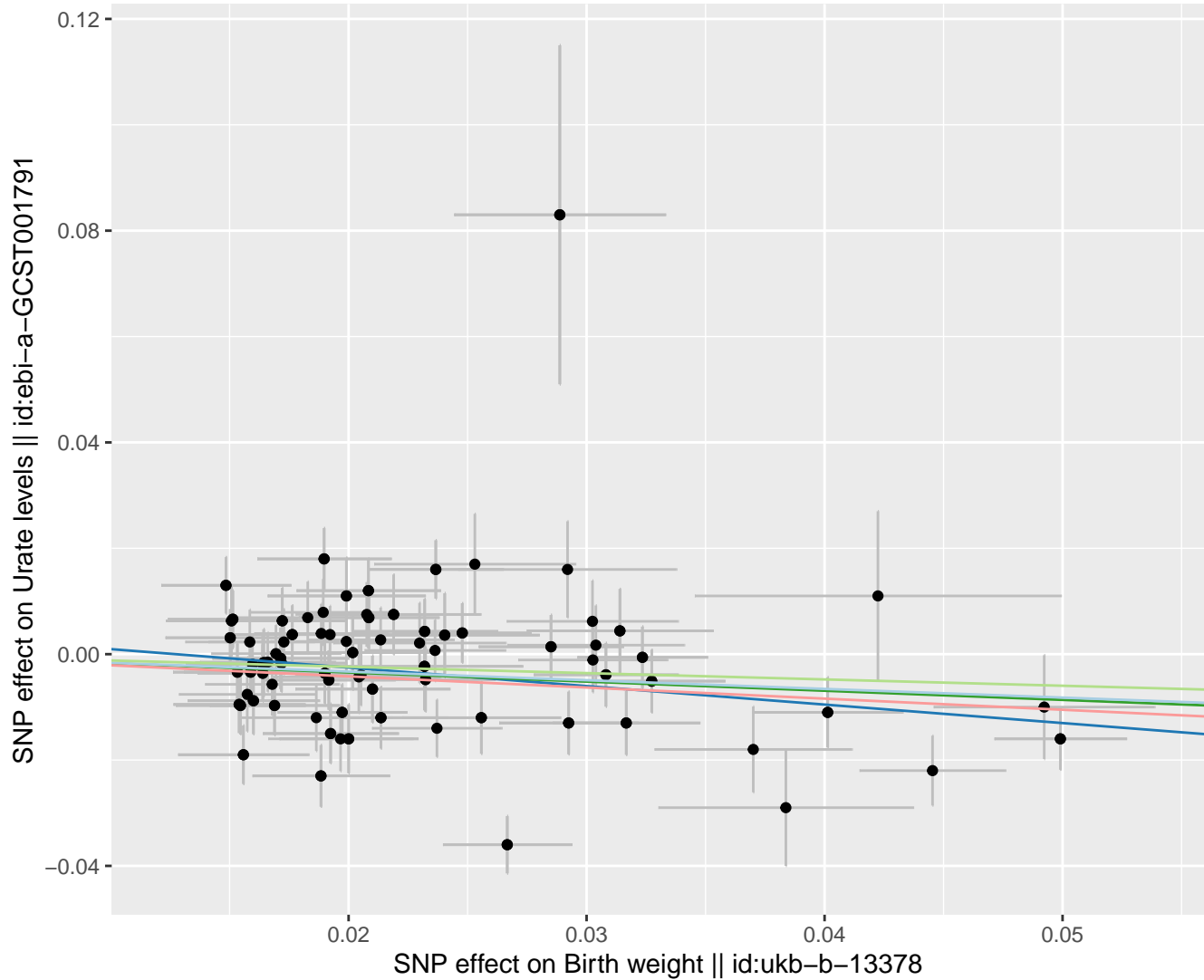

Supplement: Supplementary file 2 [file medi-105-e49237-s002.pdf]

# MR Method

- Inverse variance weighted
- MR Egger

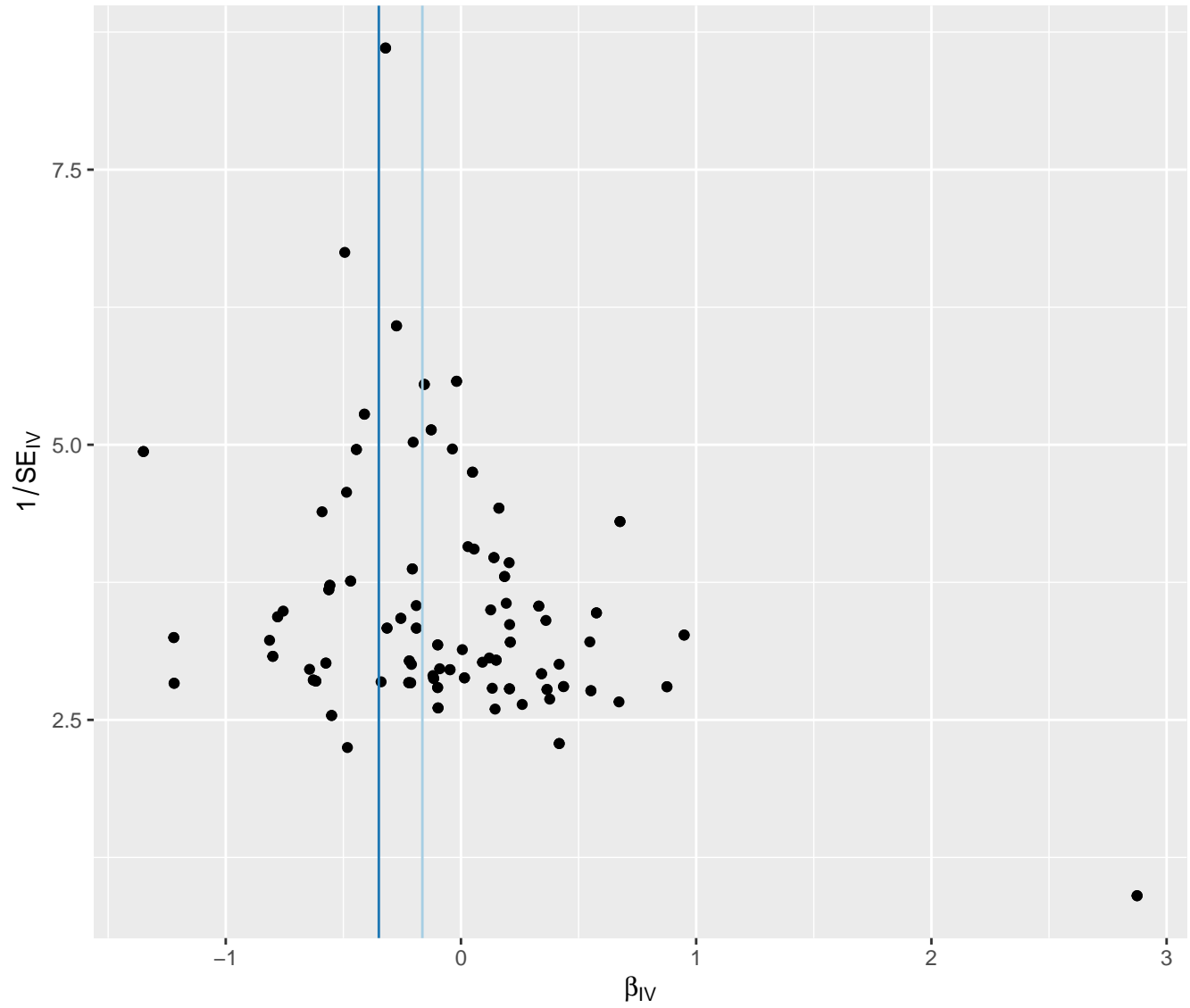

Supplement: Supplementary file 3 [file medi-105-e49237-s003.pdf]

# MR Test

- Inverse variance weighted
- MR Egger
- Simple mode
- Weighted median
- Weighted mode

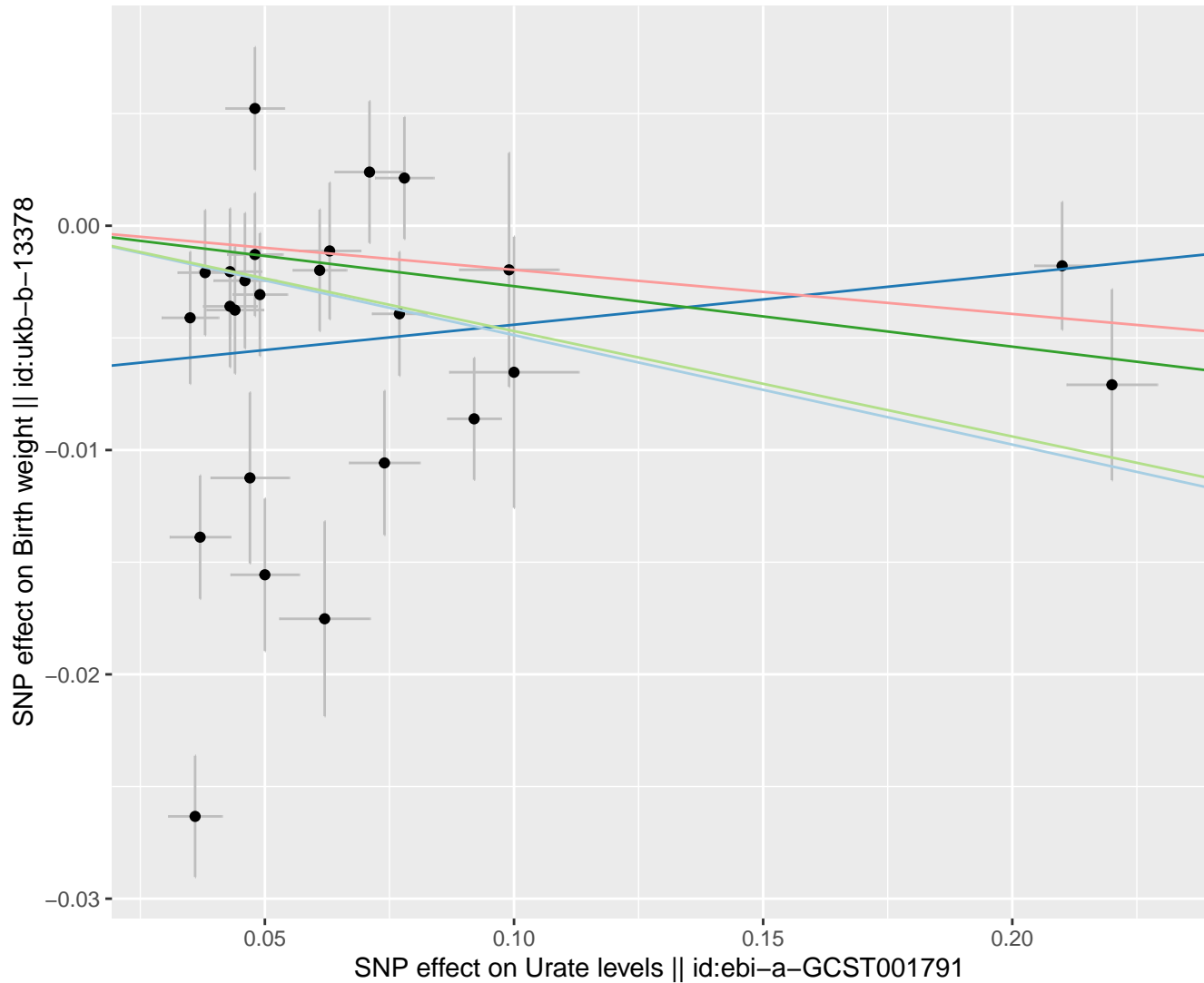

Supplement: Supplementary file 4 [file medi-105-e49237-s004.pdf]

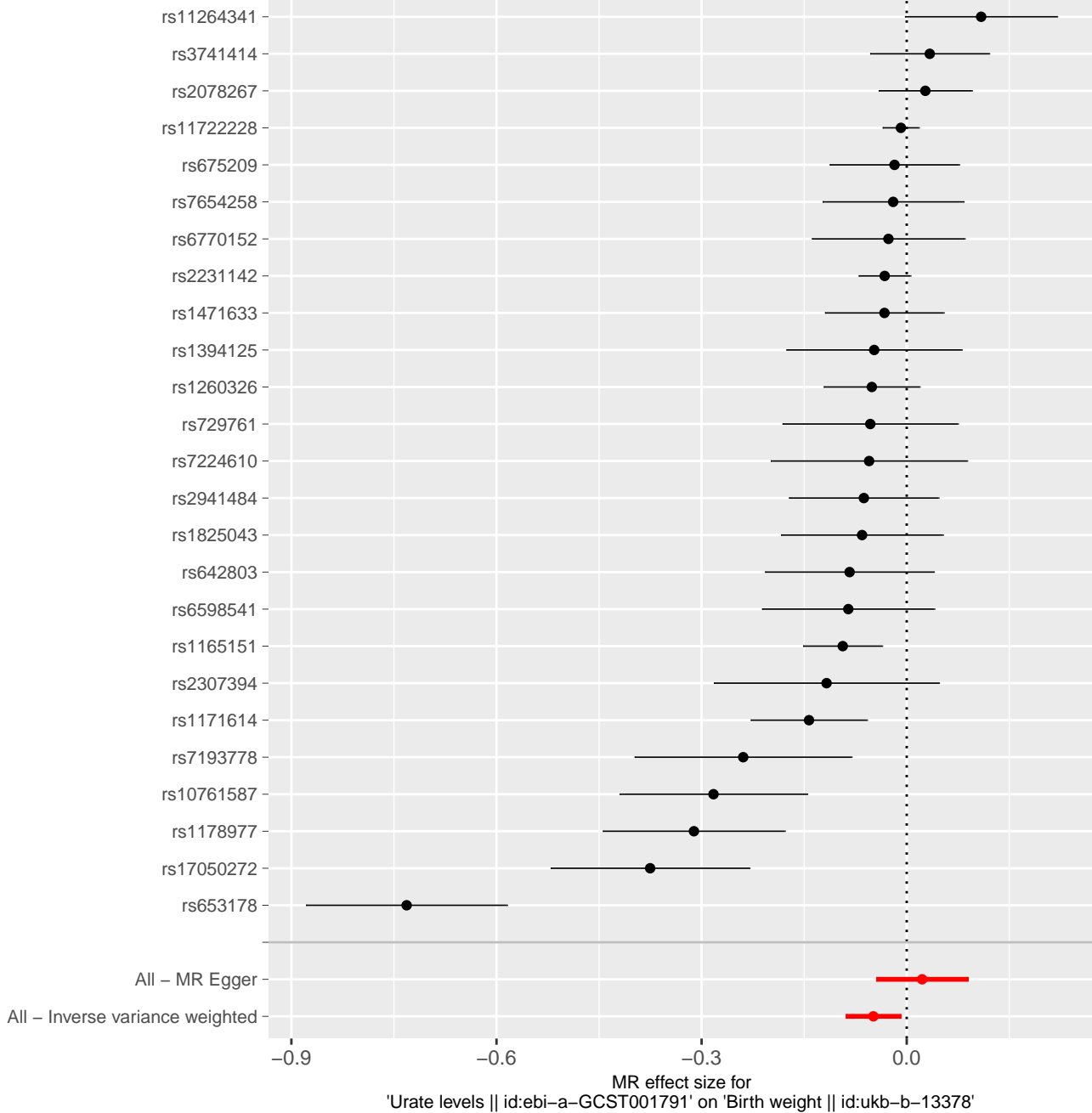

Supplement: Supplementary file 5 [file medi-105-e49237-s005.pdf]

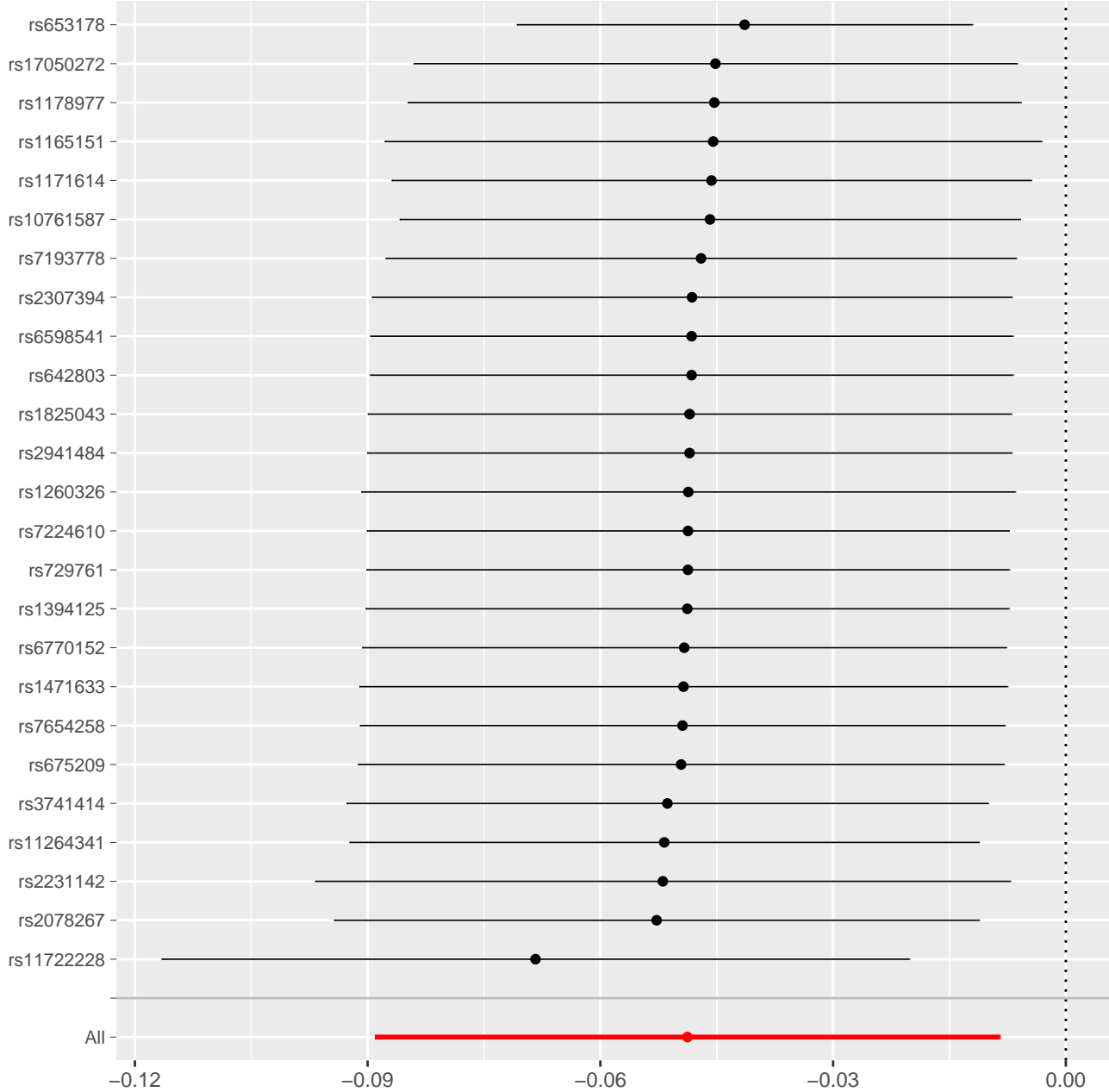

MR leave-one-out sensitivity analysis for  
'Urate levels || id:ebi-a-GCST001791' on 'Birth weight || id:ukb-b-13378'

Supplement: Supplementary file 6 [file medi-105-e49237-s006.pdf]

# MR Method

- Inverse variance weighted
- MR Egger

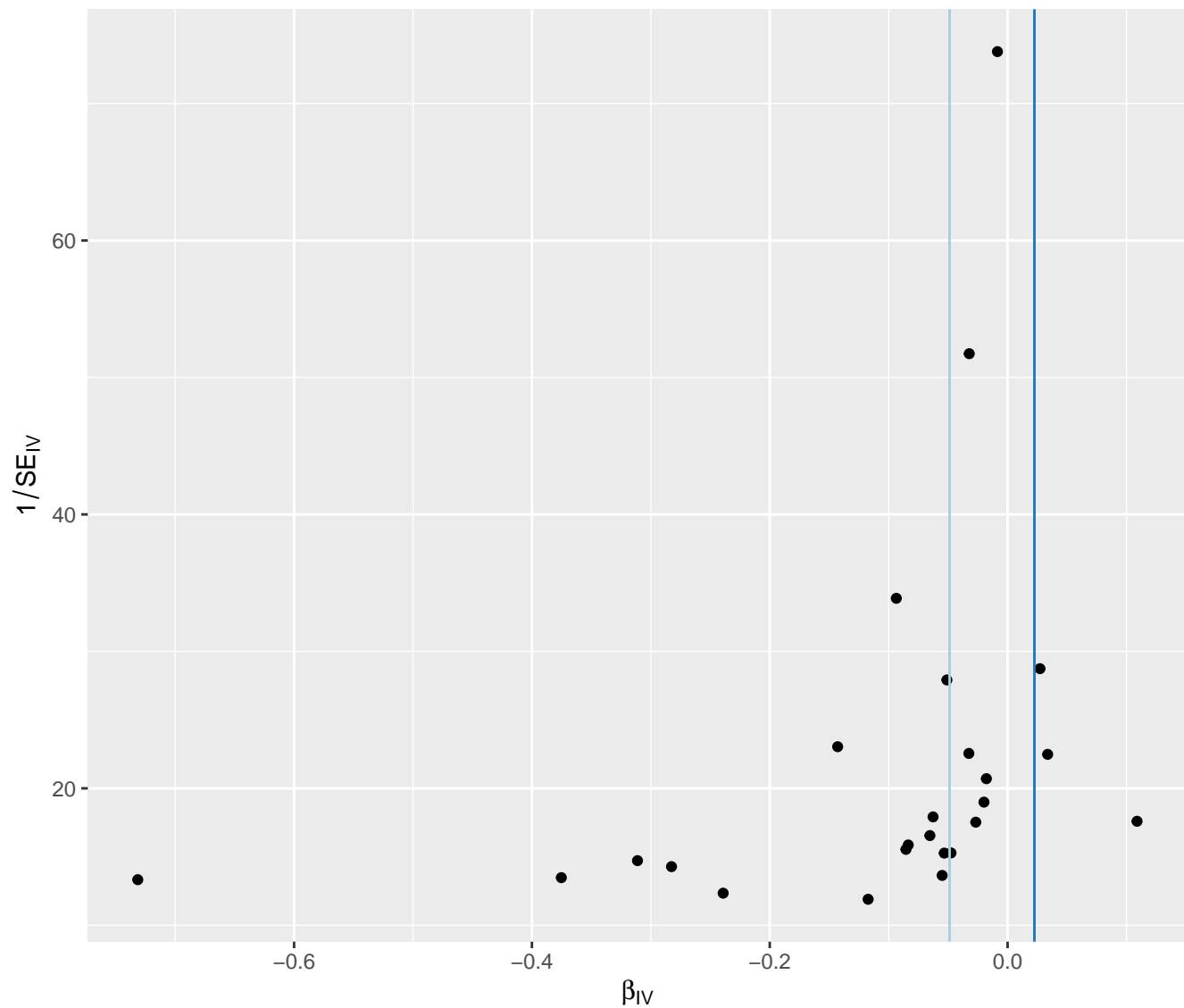

Supplement: Supplementary file 7 [file medi-105-e49237-s007.pdf]

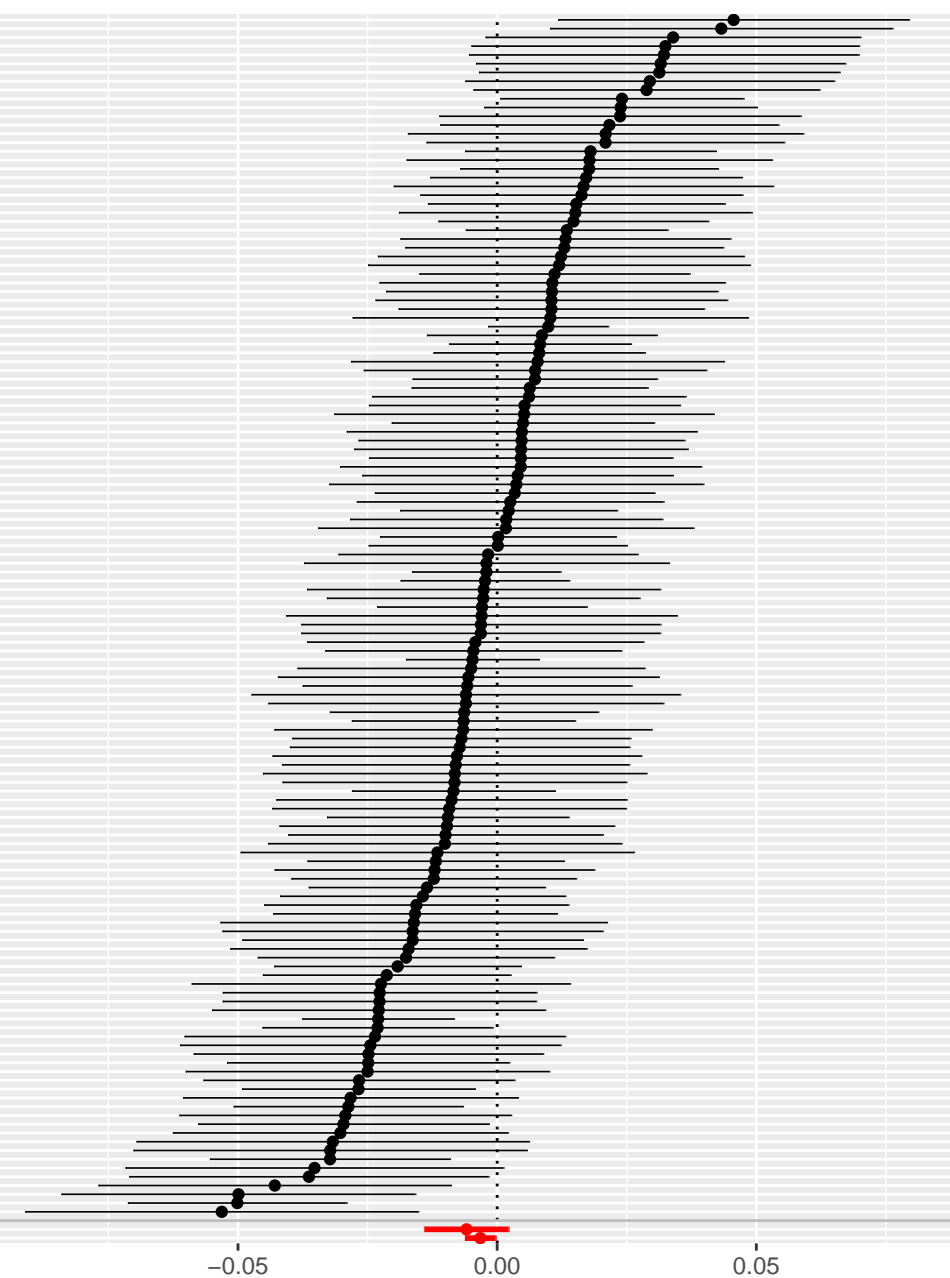

MR effect size for

Supplement: Supplementary file 9 [file medi-105-e49237-s009.pdf]

# MR Method

- Inverse variance weighted
- MR Egger

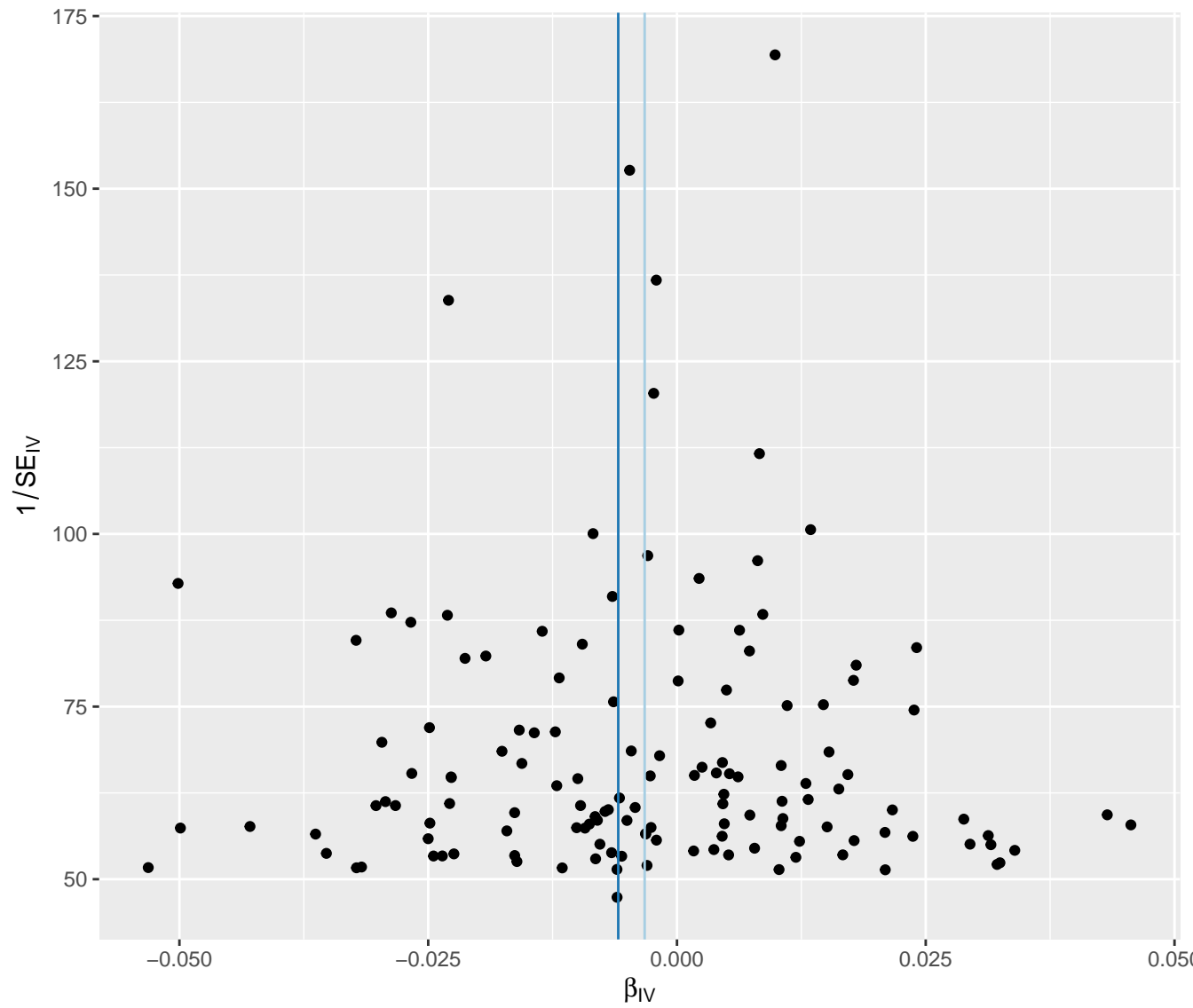

Supplement: Supplementary file 11 [file medi-105-e49237-s011.pdf]

# MR Test

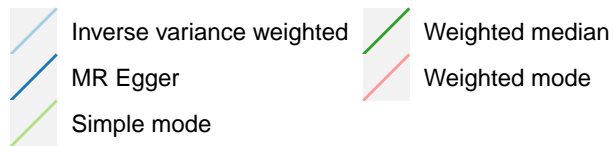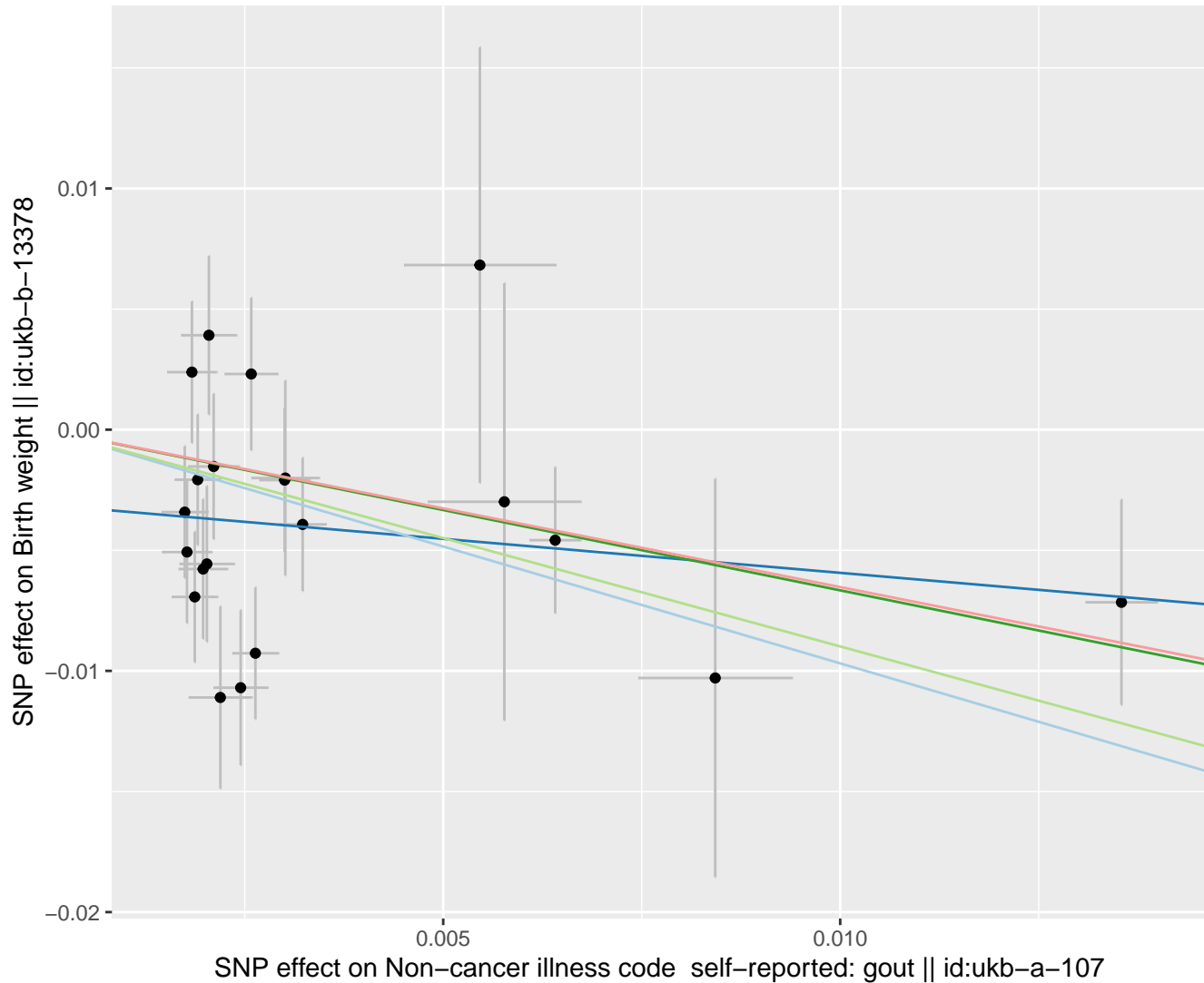

Supplement: Supplementary file 12 [file medi-105-e49237-s012.pdf]

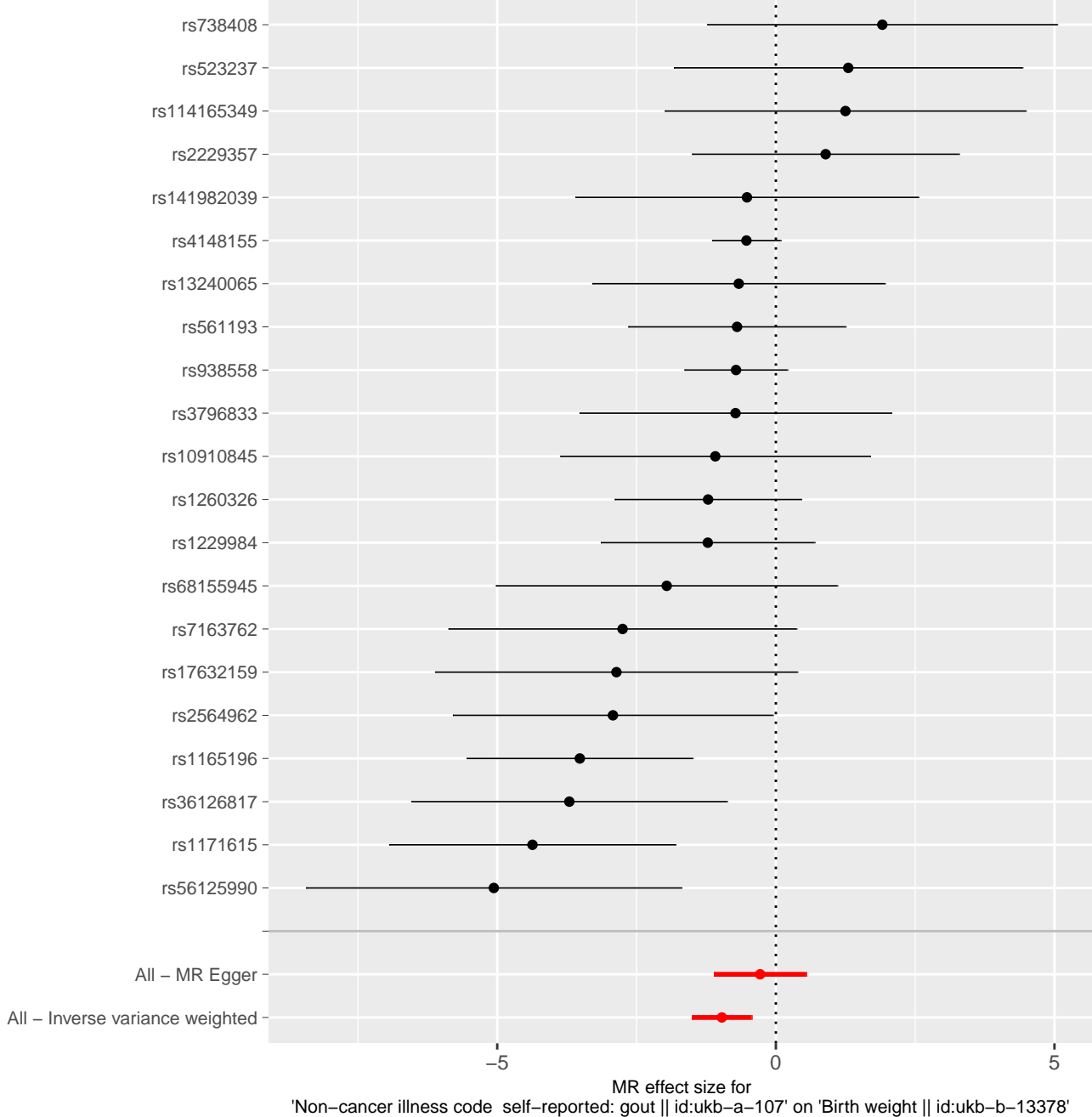

Supplement: Supplementary file 13 [file medi-105-e49237-s013.pdf]

# MR Method

- Inverse variance weighted
- MR Egger

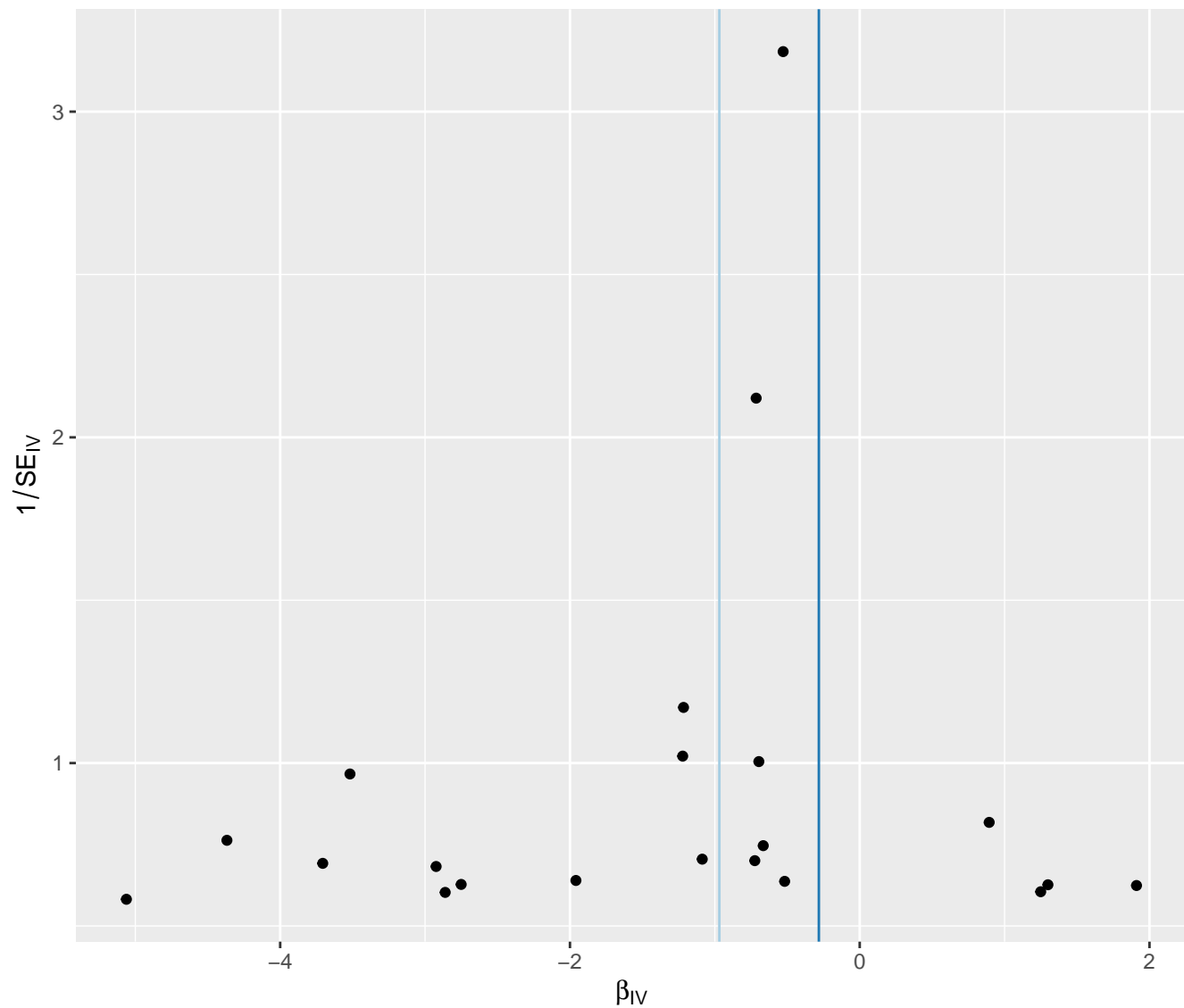

Supplement: Supplementary file 15 [file medi-105-e49237-s015.pdf]
