# Supplementary material for: Causal relationship between birth weight, uric acid levels, and risk of gout: New insights from a bidirectional two-sample Mendelian randomization study
Source: Medicine (Baltimore). 2026 Jun 5;105(23):e49237. doi: 10.1097/MD.0000000000049237 (PMC13246114; doi:10.1097/MD.0000000000049237)
Supplement: Supplementary file 8 [file medi-105-e49237-s008.pdf]

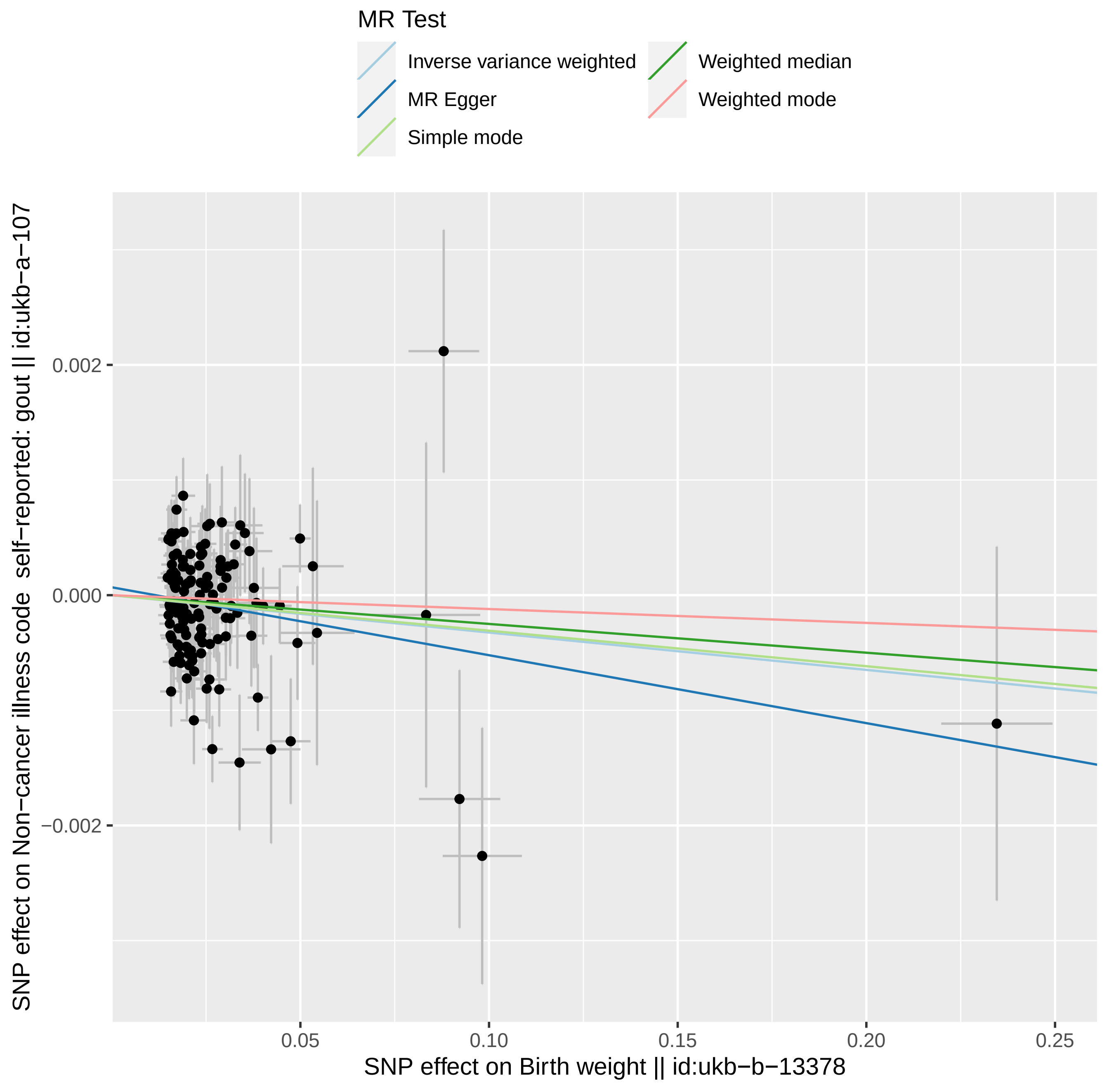

MR Test
Inverse variance weighted
MR Egger
Simple mode
Weighted median
Weighted mode
0.002
0.000
−0.002
0.05
0.10
0.15
0.20
0.25
SNP effect on Birth weight || id:ukb−b−13378
SNP effect on Non−cancer illness code self−reported: gout || id:ukb−a−107
