# Supplementary material for: Causal relationship between birth weight, uric acid levels, and risk of gout: New insights from a bidirectional two-sample Mendelian randomization study
Source: Medicine (Baltimore). 2026 Jun 5;105(23):e49237. doi: 10.1097/MD.0000000000049237 (PMC13246114; doi:10.1097/MD.0000000000049237)
Supplement: Supplementary file 14 [file medi-105-e49237-s014.pdf]

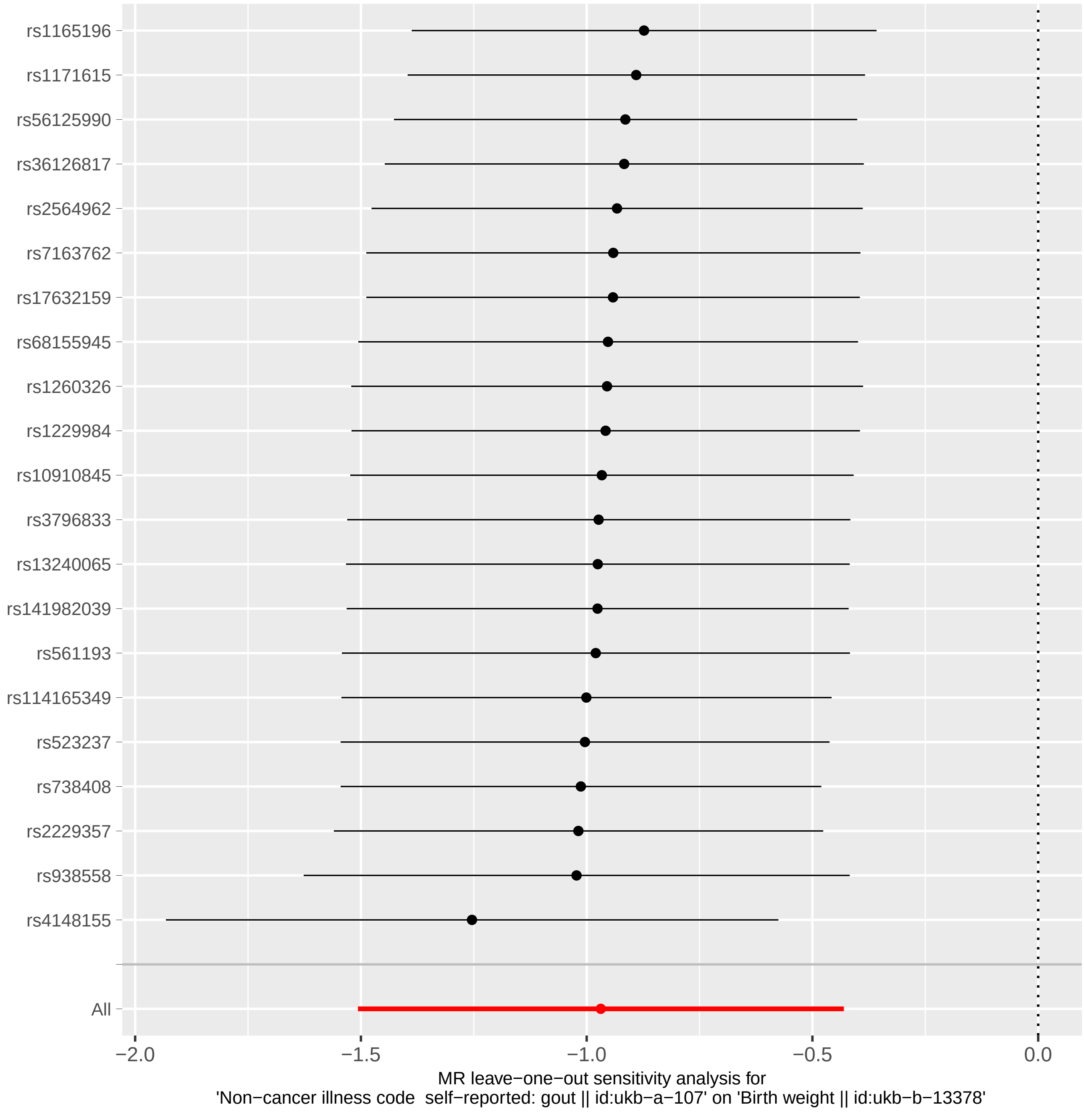
rs1165196
rs1171615
rs56125990
rs36126817
rs2564962
rs7163762
rs17632159
rs68155945
rs1260326
rs1229984
rs10910845
rs3796833
rs13240065
rs141982039
rs561193
rs114165349
rs523237
rs738408
rs2229357
rs938558
rs4148155
All
−2.0
−1.5
−1.0
−0.5
0.0
MR leave−one−out sensitivity analysis for
'Non−cancer illness code self−reported: gout || id:ukb−a−107' on 'Birth weight || id:ukb−b−13378'
