# Supplementary material for: Causal relationship between birth weight, uric acid levels, and risk of gout: New insights from a bidirectional two-sample Mendelian randomization study
Source: Medicine (Baltimore). 2026 Jun 5;105(23):e49237. doi: 10.1097/MD.0000000000049237 (PMC13246114; doi:10.1097/MD.0000000000049237)
Supplement: Supplementary file 10 [file medi-105-e49237-s010.pdf]

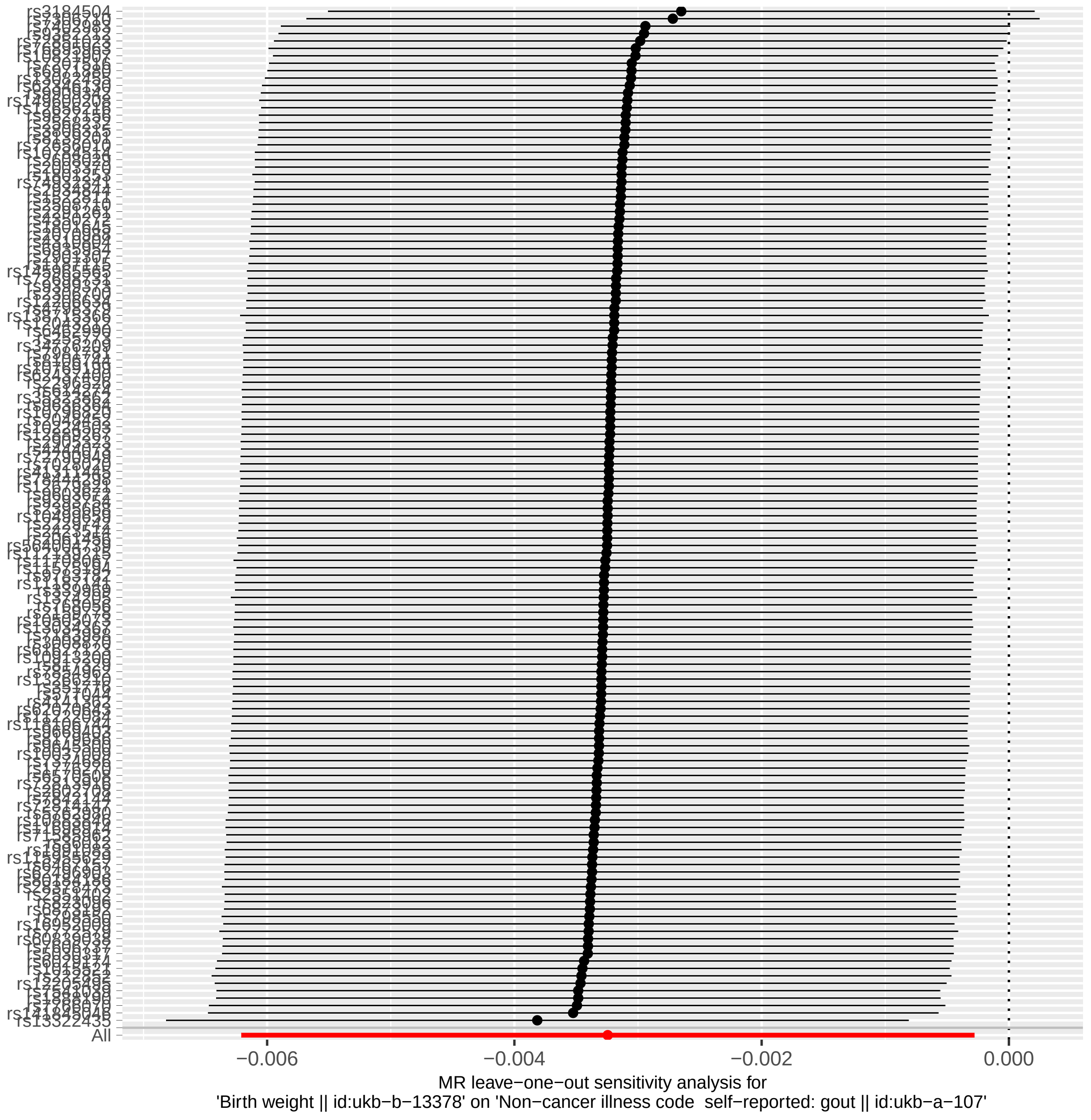
rs3184504
rs13322435
All
−0.006
−0.004
−0.002
0.000
MR leave−one−out sensitivity analysis for
'Birth weight || id:ukb-b−13378' on 'Non−cancer illness code self−reported: gout || id:ukb−a−107'
